# Change of Diet, Plasma Lipids, Lipoproteins, and Fatty Acids during Ramadan: A Controversial Association of the Considered Ramadan Model with Atherosclerosis Risk

**DOI:** 10.3329/jhpn.v29i5.8902

**Published:** 2011-10

**Authors:** Ahmed Barkia, Kamel Mohamed, Maha Smaoui, Nouri Zouari, Mohamed Hammami, Moncef Nasri

**Affiliations:** ^1^Ecole Supérieure des Sciences et Techniques de la Santé, Sfax, Tunisie; ^2^Laboratoire de Génie Enzymatique et de Microbiologie, Ecole Nationale d'Ingénieurs, Sfax, Tunisie; ^3^Laboratoire de Biochimie, UR « Nutrition Humaine et Désordres Métaboliques » Faculté de, Médecine Monastir, Tunisie; ^4^Service Exploration Fonctionnelle, Hôpital Universitaire Habib Bourguiba, Sfax, Tunisie

**Keywords:** Atherosclerosis, Diet, Fasting, Fatty acids, Lipoproteins, Ramadan, Tunisia

## Abstract

Different Islamic populations have different alimentary habits, notably during Ramadan. The paper reports the change of diet, lipids, and lipoproteins produced during Ramadan in one Tunisian population. During Ramadan, the study subjects consumed more proteins, cholesterol, vitamin E (p<0.01), and polyunsaturated fatty acids (p<0.05). At the same time, they exhibited an increase in total cholesterol, low-density lipoprotein-cholesterol (p<0.01) and apoprotein B (p<0.05) and a decrease in the ratio of apoprotein AI to apoprotein B (p<0.01). All assayed saturated fatty acids were unaffected by Ramadan fasting while three unsaturated fatty acids (C18:1cis9, C18:2n-6, and C30:4n-6) increased significantly. A return to the habitual diet for a four-week period was not sufficient to restore the pre-fasting patterns. For the study subjects, Ramadan was clearly associated with a change of diet and biochemical profile but its effective impact on atherosclerosis risk was unclear, perhaps, because other non-alimentary changes ought to be considered too. Future studies considering the non-alimentary factors, such as sleep and physical activity, would be useful to clarify the contribution of dietary change in the observed modification of biological profile.

## INTRODUCTION

Fasting in Ramadan is a religious practice respected by a large majority of Muslims, and it is associated with the decrease in the number of meals and the eating pattern which are two metabolically-active parameters (1,2). Consequently, the observed change of biologic profile accompanying the fast of Ramadan is not surprising. However, examination of data revealed that the impact of Ramadan fasting on the health is not same in different populations of Muslims (2-13). In particular, its impact on the atherosclerosis risk varies from one case to another of practising Muslim populations. While fasting is accompanied with an increase of antiatherogenic biochemical parameters [high-density lipoprotein-cholesterol (HDL-cholesterol) and apolipoprotein (apo) AI] and/or a decrease of atherogenic parameters [triglycerides (TG), total cholesterol (TC), apoprotein (apo) B, and low-density lipoprotein-cholesterol (LDL-cholesterol)] in certain practising Islamic populations (3-9), it has an opposite effect (10-12) or no impact (13) on other Muslim populations. This discrepancy could be attributed to different ethnic groups and physical activity and also to consumption of different alimentary products due to different physiopathological conditions and geographical zones, or different seasons in which Ramadan takes place.

The role of dietary fat composition in relation to risk of cardiovascular diseases (CVDs) has been a subject of intense debate for some years. While high intakes of saturated fatty acids (SFAs) are known to raise LDL-cholesterol and atherosclerosis risk, intakes of polyunsaturated fatty acids (PUFAs) (n-6 and n-3) are inversely associated with CVDs (1,14,15).

Available evidence suggests that both lipoprotein oxidative modification and physiological functions, such as blood pressure and platelet aggregation and adhesion, are key mechanisms linked to the development of atherosclerosis and its associated complications, such as CVDs. These two phenomena are influenced by the high intake of PUFAs. In fact, PUFAs are, on one hand, highly sensitive to oxidation (16,17), and on other hand, these are the precursors of active substances (prostaglandins) governing the platelet function and blood pressure. Consequently, the intake of an adequate quantity and/or quality of fatty acids and antioxidant nutriments is necessary to combat atherosclerosis.

The fatty acid composition of total plasma lipids is a reliable marker of alimentary fatty acids (18,19). Then, the study of the plasma fatty acid profile could inform us about the quality of diet. Besides, the existence of a relationship between patterns of plasma fatty acid composition and arterial stiffness has been reported (20). Despite the possible impact of the study of plasma lipid composition, studies on plasma fatty acid composition are sparse. Particularly, the effect of fasting Ramadan on the composition of plasma fatty acids has never been examined.

In Tunisia, Ramadan is always associated with an increased consumption of fruits, salads, eggs, milk, and other dairy products. The changes of diet and several lipid and lipoprotein parameters, including the pattern of plasma fatty acids, have been investigated in subjects from the same area and same socioeconomic level. The findings are presented in this paper.

## MATERIALS AND METHODS

### Subjects

Initially, the number of volunteer subjects was 36 (22 males and 14 females) but only 25 (19 males and 6 females) aged 22-55 years completed the full protocol. All the subjects were from Sfax city (a coastal Tunisian city) and were the staff of the Faculty of Medicine or that of the hospital. All these subjects had a normal glycaemia and body mass index (BMI), and none of them had a history of metabolic disease in their immediate family lineage.

### Alimentary survey

Before Ramadan, the subjects provided a written description of 4-6 diets. The average of these diets was used as the habitual one. During Ramadan, twice a week, each subject recorded the approximate quantities of food eaten. The total Ramadan diet was the average of the eight recorded meals. The nutrient intakes were calculated using the Nutritionist IV Computer Analysis software (version 3.1) (Nutritionist IV Computer Analysis Program, 1994, N2 Computing, Hearst Corp. Salem, OR, USA).

### Blood-collection samples

Before midday and approximately 9-10 hours after the last meal taken by the subjects, 10 mL of blood samples were drawn by venipuncture into tubes containing 0.01 mM of ethylene diamine tetraacetic, 2 μg/mL of sodium azoture, and 5 μg/mL of gentamicin. Plasmas were obtained by centrifugation at 3,000 rpm for 15 minutes at 6-8 °C. Glycaemia was determined immediately using a glucose oxidase method. Then, the plasmas were fractionated and placed at −20 °C until they were used again. This operation was carried out four times. The first time goes from two days before Ramadan to the first day of this month. The second and third samples were taken respectively after two weeks and four weeks of Ramadan. The fourth sample was taken one month after Ramadan.

### Analysis of plasma lipids, lipoproteins, and apolipoproteins

HDL fraction was obtained by precipitation of apoB-containing lipoproteins using phosphotungstic acid (Bio-Merieux, France). The absence of apoB in this fraction was confirmed immunologically using ready commercially-available plates (Sebia, France). TG, TC, and HDL-cholesterol were quantified by standard enzymatic techniques with spectrophotometric detection. Apolipoprotein AI and B were quantified by electrophoretic immunodiffusion using ready commercially-available plates (Sebia, France).

### Plasma fatty acid profile

The fatty acid composition of lipids was extracted from the plasma following the method of Folch *et al*. (21). Samples were dried up and esterified by heating these at 80 °C for two hours with methanol containing 2% concentrated H_2_ SO_4_. The fatty acid methyl esters of each sample were injected into a Hewlett Packard HP 5890 Series II gas chromatograph (Hewlett Packard, Palo Alto, Calif) equipped with a flame ionization detector and a polar fused silica capillary column HP-Innowax with cross-linked PEG, Carbowax 20 M (30 m × 0.25 mm ID and 0.25 μm as film thickness). The oven temperature was programmed to increase from 180 °C to 250 °C at a rate of 10 °C per minute, and the injector and the detector temperature was 220 °C and 280 °C respectively. Carrier gas was nitrogen (flow rate of 1 mL/minute). Each fatty acid concentration was expressed in absolute value (g/100 g) following an internal standard method using C17:0. Three injections per sample were performed.

### Statistical analysis

Data were expressed as means (standard deviation). The average concentrations obtained in the two subject groups were compared using Student's *t*-test while the percentages were compared using the χ^2^ test. The differences were considered significant at p<0.05.

### Ethical approval

A medical committee of the Sfax University Teaching Hospital approved the study, and informed consent was obtained from each participant after a full explanation of the study.

## RESULTS

### Characteristics of study subjects

Age, BMI, and fasting glycaemia are summarized in Table 1. Glycaemia was absolutely unchanged while BMI increased from 26.4±4.2 kg/m^2^ to 27.9±1.8 kg/m^2^. One month after Ramadan, BMI decreased but remained relatively higher than the pre-Ramadan's BMI.

### Characteristics of diets

As shown in Table 2, Ramadan fasting was associated with an increase of the estimated total energy consumption (+5.6%). The intake of proteins, cholesterol, and vitamin E (p<0.01) and PUFAs (p<0.05) increased while the consumption of carbohydrates decreased (p<0.01). In addition, the ratio of PUFAs to SFAs increased significantly (p<0.05) during Ramadan.

**Table 1.** Characteristics of recruited subjects: age; BMI, and glycaemia before Ramadan, end of Ramadan, and after Ramadan

Number	25
Sex (male/female)	19/6
Mean age (range)	42 (22-55) years
BMI (kg/m ^2^): BR/ER/AR	26.4±4.2/27.9± 1.8/27.1±2.6
Glucose (mmol/L): BR/ER/AR	4.7±0.8/4.9± 0.9/4.7±0.9

AR=After Ramadan;

BMI=Body mass index;

BR=Before Ramadan;

ER=End of Ramadan

**Table 2.** Comparison of Ramadan and habitual diets: daily energy, proteins, fats, and carbohydrate contribution (% of total energy) and daily intake of different fatty acids, fibre, vitamin C, and vitamin E

Nutrient	Ramadan diet	Habitual diet
Energy (kcal/day)	2,447±329	2,585±339 NS
% of energy contributed by		
Proteins	12.2±1.8	15.6±2.0 **
Fats	34.4±9.4	37.2±10.1
Carbohydrates	52.9±4.6	47.2±2.9 **
Nutrients intake		
Cholesterol (mg/day)	250.9±125.2	529.0±147.3 **
SFA (g/day)	28.5±8.7	31.5±8.0 NS
MUFA (g/day)	47.8±13.4	49.3±13.8 NS
PUFA (g/day)	20.1±11.3	30.9±8.7 *
PUFA/SFA (%)	0.7±0.4	1.0±0.3 *
Fibre (g/day)	16.5±4.7	18.7±6.6 NS
Vitamin E (mg/day)	6.7±4.7	9.6±1.6 **
Vitamin C (mg/day)	53.7±46.9	45.3±20.3 NS

Values are expressed as means±standard error.

* p<0.05;

** p<0.01;

MUFA=Monounsaturated fatty acid;

PUFA=Polyunsaturated fatty acid;

NS=Not significant;

SFA=Saturated fatty acids

### Pattern of total plasma fatty acids

Table 3 shows that none of the quantified SFA changed even at the end of Ramadan. However, two weeks of Ramadan fasting were sufficient to increase three polyunsaturated fatty acids: C18:1cis, C18:2n-6 (p<0.05), and C20:4n-6 (p<0.01) in a significant way. No change of MUFA/PUFAs (49-50%) and of oleic acid/PUFAs (43-44%) was observed. The continuation of Ramadan practice more than two weeks did not have any additional effect. One month after Ramadan, these fatty acids were still high but the C18:1cis level did not differ significantly from its pre-fasting values.

**Table 3.** Pre-fasting, fasting, and post-fasting fatty acid patterns (μg/mL)

Parameter	BR	R (2 weeks)	R (4 weeks)	AR (4 weeks)
C14:0	11.7±6.8	13.5±6.3	13.5±6.7	13.4±6.5
C14:1	1.3±1.8	1.6±1.8	1.2±0.8	1.1±0.9
C16:0	416.0±125.5	507.0±138.2	444.2±110.5	444.4±96.0
C16:1	23.3±16.5	27.3±12.1	24.1±17.8	21.7±9.6
C18:0	124.1±30.5	139.8±35.7	132.7±25.3	121.9±25.4
C18:1 cis	351.8±94.3	453.2±143.2*	439.9±140.0*	402.6±87.2
C18:1 trans	26.5±8.9	27.4±8.9	32.4±15.1	33.2±12.0
C18:2n-6	589.8±174.4	715.8±160.2*	702.6±145.0*	680.2±62.5*
C18:3n-3	12.1±18.9	14.9±8.7	12.1±4.5	11.2±7.8
C20:0	2.8±1.6	3.8±3.1	3.4±1.6	2.3±1.6
C20:1n-9	4.6±3.7	4.9±2.5	5.0±3.8	4.6±1.7
C20:2n-6	2.4±2.9	1.3±1.2	1.1±1.0	3.5±2.9
C20:3n-6	1.3±1.7	2.0±1.1	2.2±2.5	1.5±0.8
C20:4n-6	98.1±36.5	133.5±41.9**	146.2±51.9**	126.0±79.5*
C22:0	16.2±8.4	21.3±11.4	18.5±10.7	14.6±3.3
C22:6n-3	114.1±49.2	128.3±36.5	134.0±89.7	126.1±48.8
C24:0	16.1±7.9	28.1±20.0	22.6±10.0	25.1±20.4

Values are expressed as means±standard error. Pre-fasting sampling was done in period included between two days before Ramadan and the first day of Ramadan. The two fasting times and after fasting values were compared with the pre-fasting values.

*p<0.05;

**p<0.01;

AR=Post-fasting;

BR=Pre-fasting;

R=Fasting

### Lipids, lipoproteins, and apolipoprotein profile

TC, TG, HDL-cholesterol, LDL-cholesterol, apoAI, and apoB were quantified, and the HDL-cholesterol/LDL-cholesterol and apoAI/apoB ratios were calculated. The results are presented in Table 4. Two weeks of Ramadan diet did not have any effect on the parameters of lipids and lipoproteins studied but fasting for one month altered the plasma lipid and lipoprotein profile significantly. In fact, the concentrations of TC, LDL-cholesterol (p<0.01), and apoB (p<0.05) increased. Moreover, the apoAI/apoB and HDL-cholesterol/LDL-cholesterol values decreased. However, while the first ratio varied significantly (p<0.05), the variation of the second one was not significant (p<0.1). One month after Ramadan, the lipids and lipoprotein profile had not normalized, and they showed the same change observed at the end of Ramadan.

**Table 4.** Pre-fasting, fasting, and post-fasting plasma concentrations of lipids, lipoproteins and apolipoproteins

Parameter	BR	R (2 weeks)	R (4 weeks)	AR (4 weeks)
TC	4.4±0.5	4.5±0.6	5.0±0.7**	4.9±0.9*
TG	1.1±0.5	1.1±0.5	1.1±0.3	1.1±0.6
HDL	1.0±0.2	1.0±0.2	1.0±0.3	1.0±0.2
LDL	2.9±0.5	2.9±0.9	3.5±0.8**	3.4±1.0*
apoAI	1.4±0.2	1.4±0.2	1.3±0.2	1.3±0.3
apoB	0.8±0.2	0.9±0.2*	0.9±0.1*	0.8±0.1
HDL/LDL	0.4±0.2	0.3±0.2	0.3±0.1	0.3±0.1
apoAI/apoB	1.8±0.3	1.7±0.3*	1.5±0.4*	1.6±0.3*

Values are expressed as means±standard error. Pre-fasting sampling was done in period included between two days before Ramadan and the first day of Ramadan. After fasting, sampling was done 4 weeks after Ramadan. The two fasting-times and after-fasting values were compared with the pre-fasting values;

*p<0.05;

**p<.01;

apo=apolipoprotein;

AR=Post-fasting;

BR=Pre-fasting;

CT=Total cholesterol;

HDL=High-density lipoprotein;

LDL=Low-density lipoprotein;

R=Fasting;

TG=Triglycerides

## DISCUSSION

Dietary recommendations specify that the intakes of carbohydrate, proteins, and fats should provide 50-55%, 15%, and 30% of daily energy respectively. Also, an intake of fats composed of 50% of MUFAs, 25% of PUFAs, and 25% of SFAs was recommended. Despite the minor differences, the habitual and Ramadan diets of this study were concordant with these recommendations. However, fewer carbohydrates and more proteins were consumed during Ramadan (p<0.01). Also, the Ramadan diet was characterized by higher levels of cholesterol, PUFAs, and vitamin E. The higher intakes of PUFAs and vitamin E could be attributed to the higher consumption of raw vegetable oil, especially olive oil, which has high vitamin E (present at 20-75 mg/100 g of olive oil; unpublished results) and PUFAs. Since cholesterol comes from animal lipids which are particularly rich in SFAs, the absence of change of these acids concomitantly to cholesterol is remarkable. This contrast could be explained by the high consumption of certain alimentary products with a particular composition as the briks (fried eggs). Egg contains a high level of cholesterol, and it has more unsaturated fatty acids (UFAs) than SFAs (about two-thirds UFAs vs one-third SFAs) (22,23).

In agreement with previous observations (2-12), fasting Ramadan causes a change of biological and anthropometric profiles. At the end of Ramadan, our subjects exhibited an increase in BMI. This variation was not significant but it could be a great change of major clinical significance. In fact, if every person gained +1 BMI in Ramadan of each year, the whole population would be obese. Fortunately, the BMI is probably normalized after Ramadan as indicated by its relative decrease one month after Ramadan.

A diet high in cholesterol and in fatty acids is associated with an increased risk of atherosclerosis. However, the real impact of alimentary fatty acids appears according to their nature and respective levels. The SFAs and trans-fatty acids (TFAs) are recognized favourable to CVDs while the PUFAs are generally considered antiatherogenic (24-28). However, the latter fatty acids have a complex relationship with atherosclerosis. In particular, they become atherogenic factors if they are consumed at a disproportional quantity compared to SFAs and/or insufficiently protected against the oxidative process. The maximal beneficial action of PUFAs was reported for the PUFA/SFA ratio=1 (29). In connection with that, the Ramadan diet of the study subjects appears to be positive: first, because the PUFA/SFA ratio increased, and it went very close to the recommended value, and second, because of its improved potential of protection against the oxidative deterioration as suggested by the increase of vitamin E intake.

Several studies reported that the rates of various fatty acids in plasma reflected the rate in food (30,31). Consistent with these data, we found that levels of the PUFA and PUFA/SFA ratio varied similarly in the diet and plasma. The increase in PUFAs was primarily due to one of w6 which could express an increased consumption of raw vegetable oils, mainly olive oil. Consistent with this idea, oleic acid also increased.

It is commonly admitted that the high intake of alimentary SFAs, TFAs, and cholesterol is associated with an increase in total cholesterol, LDL-cholesterol, and apoB concentration and/or decrease of HDL-cholesterol (24-27,32). As it was shown in the composition diets (Table 2), the intake of SFAs was unvaried in Ramadan. The total content of TFA in the diet was not determined and only C18:1trans was quantified in plasma. However, the fact that this TFA plasma level did not change during Ramadan, and the fact that our subjects did not consume more processed foods which are rich in TFA (as margarine) suggests that the intake of TFAs did not vary significantly during Ramadan. So, the increase of TC, LDL-cholesterol, and apoB and the reduction of apoAI/apoB ratio (Table 3) observed in our subjects during Ramadan could be attributed mainly to the high intake of cholesterol (33). The consumption of such amounts of cholesterol was previously associated with the formation of abnormal and cholesterol-enriched LDL (17). This does not seem to be the case in the study subjects because LDL-cholesterol/apoB was unchanged. Furthermore, we had not observed the reduction of TC and TC/HDL-cholesterol ratio in response to high intakes of PUFAs (4,34) perhaps because of a higher hypercholesterolaemic effect of the consumed cholesterol. However, an inadequate quantity and/or quality of alimentary fatty acids must be considered.

The plasma lipid profile and CVD risk in mice are said to be sensitive to the diet ratio of ω 6 PUFA to eicosapentaenoic acid (EPA, C20:5ω 3) and docosahexaenoic acid (DHA, C22:6ω 3) [ω 6/(EPA+DHA)] (35,36). Both n-6 and n-3 PUFAs serve as precursors of other important compounds. Excess of one family of fatty acids can interfere with the metabolism of the other and its overall biological effects.

As is suggested by the increase of the ω 6/ω 3 ratio (5.46 before Ramadan and 5.84-5.96 during Ramadan) in plasma lipids which reflects alimentary lipids (18,19), Ramadan diet could have an unfavourable PUFA composition. Essentially, Ramadan is associated with an increased consumption of vegetable oil and a decrease in fish consumption.

The increase of n-6 PUFA in plasma lipids was justified by the concomitant increase of linoleic and arachidonic acids. The increase of these two acids was not surprising because they are metabolically linked (linoleic acid is the precursor of arachidonic acid). However, we must emphasize the more pronounced change of the second fatty acid as suggested by the more significant difference between its habitual and its Ramadan values (p<0.01 vs p<0.05 for C18:2n-6) and by the decrease of the C18:2n-6/C20:4n-6 ratio which fell from about 6 before Ramadan to about 4.9 in Ramadan. Such an observation could be attributed, at least in part, to the activation of the linoleic acid to arachidonic acid, especially the absence of linolenic acid (C18:3 n-3), which is able to inhibit this conversion (37).

Now, it is well-known that the atherosclerotic process involves many and various mechanisms which are influenced by lipids or their derivatives. For example, the pattern of fatty acids (FAs) could affect the atherosclerosis risk by affecting the blood fluidity which depends on the production of prostaglandins obtained from arachidonic acid (ω 6). Also, the FA pattern, particularly the level of AA, has been reported to be important for the elasticity of arteries (38). An observed increase of n-6 in plasma could signify an adequate production of prostaglandins and correct elasticity of arteries and, then, a decrease of both blood clotting and calcification of arteries. On the other hand, high levels of PUFAs could be favourable to the atherogenic process. For example, they could stimulate the formation of adipocyte as it was in mouse (39) and, consequently, induce obesity, which is associated with a high risk of CVDs. Also, because PUFAs are highly sensitive to the oxidation process, the increase of their levels could be associated with an increased oxidative modification of LDL which becomes more atherogenic (40). However, this situation was not obligatory in our subjects because the concomitant higher intake of vitamin E could lead to a relative protection. In addition, in recent work, Mauerer *et al*. observed that linoleic acid reduced the ABCA1 and ABCG1 expression in M-CSF predifferentiated macrophages, and they concluded that it was associated with an increase of the formation of atherosclerotic lesions by diminishing the reverse cholesterol transport function of ABCA1 and ABCG1 (41). Finally, PUFAs affect the expression of genes controlling the inflammatory process which characterizes the atherosclerosis. In this respect, the n-6 and n-3 families of PUFAs appear to have opposing and antagonistic effects. While n-3 PUFA increases the cells resistant to inflammation by eliminating the expression of inflammatory genes, n-6 PUFA exerts the opposite effect (42).

Considering the total data, the ratio of n-3 PUFA to n-6 PUFA appears as a determining factor for the protective role which has been proposed as an important index of cardiovascular risk. Based on the plasma fatty acid profile, this ratio may suffer an adverse change in Ramadan.

The return to habitual diet tends to restore the normal lipid and lipoprotein profile. However, the change, caused by Ramadan fasting, does not disappear four weeks after. It is possible that some eating and/or non-eating habits in Ramadan persist for some time later. A study of subjects during a longer time after Ramadan could have been very beneficial but it was difficult to retain the studied subjects for a longer period.

The interpretation of our results is delicate. Although the high intake of both vitamin E and PUFAs could have beneficial effects, the change of plasma lipid and lipoprotein profile was atherogenic (increase of plasma TC, LDL-cholesterol, apoB, and ω 6/ω 3).

In this paper, we provide evidence for the complexity of the relationship between Ramadan diet and the health state, particularly with atherosclerosis risk. To know the real effect of our subjects' Ramadan fasting model, it is notably necessary to study other parameters [Lp(a), apoAI-containing lipoproteins, TFAs, oxidant status, LDL size, etc.] and physiological functions (blood coagulation, platelets function, apoB-receptor activity, cholesterol efflux, etc.).

The knowledge about the effective impact of Ramadan fasting could be of great interest for possible intervention to correct the Ramadan diet of certain populations of practising Muslims.

The comparison of the dietary changes in different Islamic populations and of their impact on various biological and biochemical parameters may be a valid approach in determining a diet model to be followed by Muslims and to advise even non-Muslims.

## ACKNOWLEDGEMENTS

This work was funded by the Ministry of High Education and Scientific Research, Tunisia. The authors thank all the volunteers who participated in the study. They also thank the hospital staff of the Centre Hospitalo-Universitaire, Habib Bourguiba of Sfax, for collecting the blood samples.
